# Pd/C-mediated synthesis of α-pyrone fused with a five-membered nitrogen heteroaryl ring: A new route to pyrano[4,3-*c*]pyrazol-4(1*H*)-ones

**DOI:** 10.3762/bjoc.5.64

**Published:** 2009-11-11

**Authors:** Dhilli Rao Gorja, Venkateswara Rao Batchu, Ashok Ettam, Manojit Pal

**Affiliations:** 1 Institute of Science and Technology, JNT University, Kukatpally, Hyderabad 500072, India; 2 Dr. Reddy’s Laboratories Ltd, Bollaram Road, Miyapur, Hyderabad 500049, India; 3 New Drug Discovery, R&D Center, Matrix Laboratories Ltd, Anrich Industrial Estate, Bollaram, Jinnaram Mandal, Medak District, Andra Pradesh 502 325, India; 4 present address: Institute of Life Sciences, University of Hyderabad Campus, Gachibowli, Hyderabad 500 046, Andhra Pradesh, India

**Keywords:** C–C bond, catalysis, palladium, pyrazole, pyrone

## Abstract

Pd/C-mediated alkynylation of 5-iodo-pyrazole-4-carboxylic acid, involving the first regioselective construction of α-pyrone ring on a pyrazol moiety via tandem coupling–cyclization process, has been developed to afford pyrano[4,3-*c*]pyrazol-4(1*H*)-one in a single pot.

## Introduction

**Figure 1:**
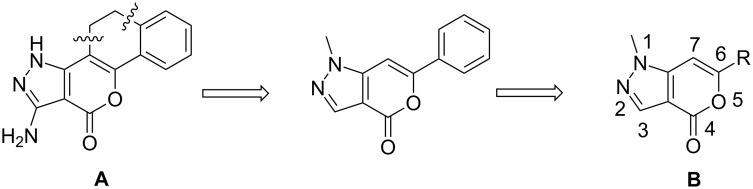
Polycyclic azaheteroaromatics (**A**) and pyrano[4,3-*c*]pyrazol-4(1*H*)-ones (**B**).

α-Pyrones [1–2] (or 2*H*-pyran-2-one) and their benzo derivatives, e.g. isocoumarins [3], have shown a wide range of pharmacological activities [4–6] such as antifungal, antimicrobial, phytotoxic and other effects. On the other hand α-pyrones fused with a five-membered heteroaryl ring, e.g. thienopyranones, have shown anticancer properties in vitro [7]. Recently, incorporation of another five-membered ring, e.g. pyrazole pyrone moieties in a single molecule (**A**, Figure 1) has been reported to provide polycyclic azaheteroaromatics with a steroid-like skeleton [8]. This initiative was based on the assumption that both the pyrazole and the pyrone moiety would be responsible for enhanced anabolic activity of the individual parent compounds. Because of our longstanding interest in the synthesis of pyrone derivatives of potential pharmacological interest we decided to explore the synthesis of α-pyrones fused with a pyrazole ring, e.g. pyrano[4,3-*c*]pyrazol-4(1*H*)-one **B** (Figure 1).

**Scheme 1:**
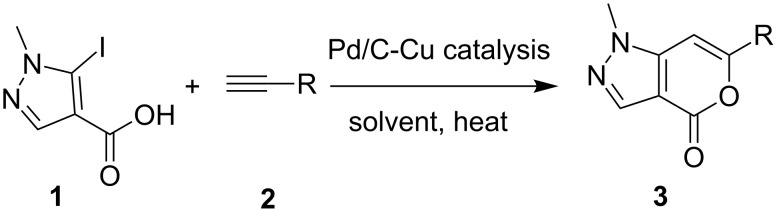
Pd/C-mediated synthesis of 6-substituted pyrano[4,3-*c*]pyrazol-4(1*H*)-ones **3**.

Synthesis of compound **B** could be achieved by either of the following strategies: construction of the six-membered ring followed by five-membered one or vice versa. However, only one method has been reported so far that falls to the first category. It involves the reaction of methyl 2-cyano-3,3-dimethylthioacrylate with 1-tetralone and subsequently with hydrazine [8]. Though a number of methods are known for the synthesis of isocoumarins by constructing the pyrone ring [9–17] (the second strategy) none has been reported for the class of compounds represented by **B**. One of the common methods used for the construction of a pyrone ring fused with benzene or with other heteroaryl moieties is Sonogashira-type coupling followed by electrophilic or transition metal mediated cyclization of the resulting alkyne (possessing a carboxylate or an equivalent group in proximity to the triple bond). Thus, isocoumarins have been prepared by reacting *o*-iodobenzoic acid with terminal alkynes in the presence of a Pd-catalyst [14–16]. Recently, we have reported synthesis of thienopyranones following a similar strategy [7,18]. On the other hand construction of a pyridine ring on a pyrazole moiety has been described under Ni or Pd catalysis [19]. Herein we report the first construction of an α-pyrone ring on a pyrazol moiety leading to the synthesis of 6-substituted pyrano[4,3-*c*]pyrazol-4(1*H*)-one **3** under Pd/C-Cu catalysis (Scheme 1).

## Results and Discussion

**Scheme 2:**
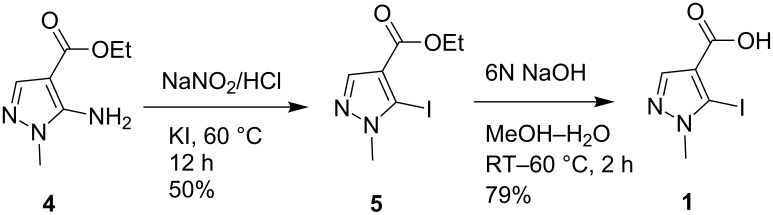
Preparation of 5-iodo-1-methyl-1*H*-pyrazole-4-carboxylic acid (**1**).

The starting material required for our synthesis, 5-iodo-1-methyl-1*H*-pyrazole-4-carboxylic acid (**1**), was prepared from commercially available ethyl 5-amino-1-methyl-1*H*-pyrazole-4-carboxylate [20] (**4**) via a two-step process as shown in Scheme 2. Thus Sandmeyer type reaction of amine **4** to introduce the iodo group on the pyrazole ring followed by hydrolysis of the ester **5** yielded the desired acid **1**.

Our study started with the reaction of iodo acid **1** with a terminal alkyne, e.g. 1-hexyne (**2a**) and the results are summarized in Table 1. Due to our earlier success in the use of 10% Pd/C-PPh_3_-CuI as a catalyst system [21] for coupling-cyclization reactions we decided to conduct the initial reaction of **1** (1.0 equiv) with **2a** (2.0 equiv) in the presence of same catalysts in ethanol using Et_3_N as a base. The reaction proceeded smoothly affording the desired product **3a** in 72% yield (Entry 1, Table 1) and no isomeric compound, i.e. 1-methyl-6-pentylidene-1*H*-furo[3,4-c]pyrazol-4(6*H*)-one, was isolated. The reaction was carried out for 16 h and increase of reaction time did not improve the product yield (Entry 2, Table 1). Product formation was almost suppressed in the absence of PPh_3_ (Entry 3, Table 1). The use of other catalysts, e.g. PdCl_2_(PPh_3_)_2_, Pd(PPh_3_)_4_ or Pd(OAc)_2_-PPh_3_, afforded the product **3a** in inferior yield (Entries 4-6, Table 1). Ethanol was found to be the best solvent in our study as the use of other solvents, e.g. 1,4-dioxane or DMF, decreased the product yield (Entries 7 and 8, Table 1). Finally, the use of fewer equiv of **2a** also decreased the product yield significantly (Entry 9, Table 1) perhaps due to the evaporation of **2a** under the conditions employed.

**Table 1:** Effect of reaction conditions on the Pd-catalyzed coupling of 5-iodo-1-methyl-1*H*-pyrazole-4-carboxylic acid (**1**) with 1-hexyne (**2a**).^a^

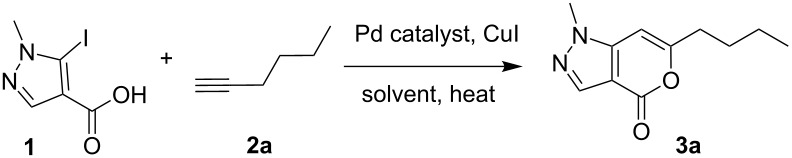			
Entry	Catalyst	Solvent; time	Yield (%)^b^

1.	10% Pd/C-PPh_3_	EtOH; 16h	72^c^
2.	10% Pd/C-PPh_3_	EtOH; 24h	70^c^
3.	10% Pd/C	EtOH; 16h	13
4.	PdCl_2_(PPh_3_)_2_	EtOH; 16h	51
5.	Pd(PPh_3_)_4_	EtOH; 16h	55
6.	Pd(OAc)_2_-PPh_3_	EtOH; 16h	48^c^
7.	10% Pd/C-PPh_3_	1,4-Dioxane; 16h	59^c^
8.	10% Pd/C-PPh_3_	DMF; 16h	61^c^
9.	10% Pd/C-PPh_3_	EtOH; 16h	55^c,d^

^a^Reaction conditions: **1** (1.0 equiv), terminal alkyne **2a** (2.0 equiv), Pd-catalyst (0.05 equiv) or Pd/C (0.035 equiv), CuI (0.06 equiv), Et_3_N (5.0 equiv) in a solvent at 70–80 °C under N_2_. ^b^Isolated yield. ^c^PPh_3_ used: 0.3 equiv. ^d^1.5 equiv of **2a** was used.

Having prepared the pyranopyrazol-4-one **3a** successfully we decided to explore the scope and generality of this reaction in the synthesis of other analogues especially varying the substituent at C-6. Accordingly, a variety of commercially available terminal alkynes were reacted with the iodo acid **1** (Table 2) under the optimized conditions as presented in Entry 1 of Table 1. As evident from Table 2, all the terminal alkynes participated well in this coupling-cyclization reaction affording the desired products in moderate to good yields. Various substituents such as alkyl, hydroxyalkyl or aryl groups present in the terminal alkyne were well tolerated. The use of arylalkynes (Entries 7 and 8, Table 2) however led to the moderate yields of product as this class of alkynes is known to undergo rapid dimerization. Deiodination of **1** as a side reaction was also observed in these cases. All the products isolated were well characterized by spectral (NMR, IR and MS) data. The appearance of a vinylic signal in the region δ 6.3–6.8 in the ^1^H NMR spectrum and a carbonyl absorption in the region 1720–1730 cm^−1^ in the IR spectrum confirmed the presence of a pyrone ring in compound **3**. This was further supported by the C=O signal in the region 160–170 ppm in the ^13^C NMR spectrum.

**Table 2:** Pd/C-mediated synthesis of 6-substituted pyrano[4,3-*c*]pyrazol-4(1*H*)-ones^a^ (**3**).

Entry	Alkyne **2** (R =)	Time (h)	Products (**3**)		Yield (%)^b^

1.	n-butyl	16	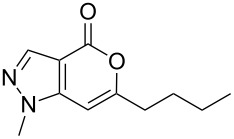	**3a**	72
2.	n-pentyl	18	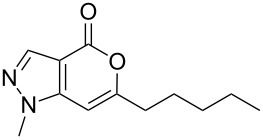	**3b**	68
3.	n-hexyl	16	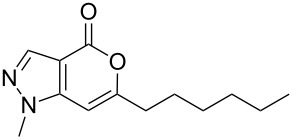	**3c**	67
4.	–CH(OH)CH_3_	12	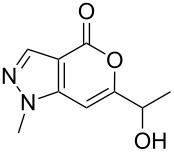	**3d**	65
5.	–C(OH)Me_2_	12	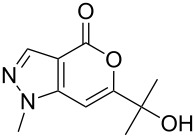	**3e**	72
6.	–(CH_2_)_3_OH	16	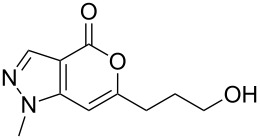	**3f**	60
7.	–C_6_H_5_	12	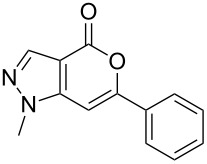	**3g**	54
8.	–C_6_H_4_CH_3_-*p*	12	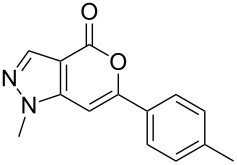	**3h**	50

^a^Reaction conditions: **1** (1.0 equiv), terminal alkyne **2** (2.0 equiv), 10% Pd/C (0.035 equiv), PPh_3_ (0.3 equiv), CuI (0.06 equiv), Et_3_N (5.0 equiv) in a solvent at 70–80 °C under N_2_. ^b^Isolated yield.

**Scheme 3:**
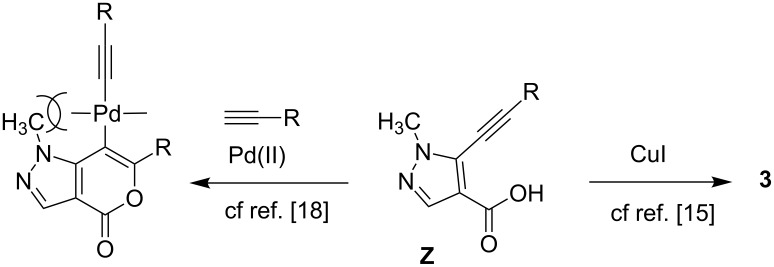
Mechanism of ring closure of intermediate alkyne **Z**.

Mechanistically, the reaction proceeds via a C–C bond forming reaction between the halide **1** and the terminal alkyne **2** in the presence of Pd(0) generated in situ. The resulting alkyne **Z** (Scheme 3) thus formed subsequently undergoes 6-*endo-dig* ring closure in an intramolecular fashion to give the desired product **3**. A favorable geometry associated with the formation of the 5-6 ring over the 5-5 ring did not allow the intermediate alkyne **Z** to undergo a 5-*exo-dig* ring closure though it is favoured by Baldwin’s rules. Unlike 3-iodo thiophene-2-carboxylic acid [18] the coupling of **1** with terminal alkynes **2** in the presence of PdCl_2_(PPh_3_)_2_ and CuI did not provide the 7-alkynyl substituted derivative of **3** possibly due to the “peri” effect caused by the N-1 methyl group of the pyrazole ring.

## Conclusions

In conclusion, we have developed a new, straightforward and general method for the regioselective synthesis of novel 6-substituted pyrano[4,3-*c*]pyrazol-4(1*H*)-ones under Pd/C-Cu catalysis, preparation of which would be difficult via other methods. The reaction proceeds via tandem C-C and C-O bond formation between the 5-iodopyrazole-4-carboxylic acid and a terminal alkyne in the same pot. Being an integral part of many drugs or bioactive molecules, the pyrazole framework plays an important role in pharmaceutical research. We therefore believe that the present methodology and the product pyranopyrazol-4-one being a potential synthon for more complex heterocycles may find wide applications in organic synthesis, especially in medicinal chemistry.

## Supporting Information

File 1 General procedure for the preparation of **3** and spectral data for selected compounds.
