# Supplementary material for: Pd/C-mediated synthesis of α-pyrone fused with a five-membered nitrogen heteroaryl ring: A new route to pyrano[4,3-c]pyrazol-4(1H)-ones
Source: Beilstein J Org Chem. 2009 Nov 11;5:64. doi: 10.3762/bjoc.5.64 (PMC2839514; doi:10.3762/bjoc.5.64)
Supplement: File 1 — General procedure for the preparation of 3 and spectral data for selected compounds. [file Beilstein_J_Org_Chem-05-64-s001.docx]

Supporting Information

**Pd/C-mediated synthesis of α-pyrone fused with a five-membered nitrogen heteroaryl ring: A new route to pyrano[4,3-*c*]pyrazol-4(1*H*)-ones**

Dhilli Rao Gorja^1^,Venkateswara Rao Batchu^2^, Ashok Ettam^2^, Manojit Pal*^3,4^

Address: ^1^Institute of Science and Technology, JNT University, Kukatpally, Hyderabad 500072, India, ^2^Dr. Reddy’s Laboratories Ltd, Bollaram Road, Miyapur, Hyderabad 500049, India, ^3^New Drug Discovery, R&D Center, Matrix Laboratories Ltd, Anrich Industrial Estate, Bollaram, Jinnaram Mandal, Medak District, Andra Pradesh 502 325, India, ^4^present address: Institute of Life Sciences, University of Hyderabad Campus, Gachibowli, Hyderabad 500 046, Andhra Pradesh, India.

Email Manojit Pal - manojitpal@rediffmail.com

*Corresponding author

**General procedure for the preparation of** **3**

A mixture of 5-iodo-1-methyl-1*H*-pyrazole-4-carboxylic acid **1** (1.0 mmol), 10% Pd/C (0.035 mmol), PPh_3_ (0.3 mmol), CuI (0.06 mmol), and triethylamine (5.0 mmol) in EtOH (10 mL) was stirred at 25–30 °C for 30 min under nitrogen and the terminal alkyne **2** (2.0 mmol) was added. The mixture was then stirred at room temperature for 1.0 h and then at 75–80 °C for the time indicated in Table 2. After completion of the reaction the mixture was cooled to room temperature, diluted with EtOAc (50 mL), and filtered through celite. The filtrate was washed with saturated aqueous sodium hydrogen carbonate (2 × 25 mL) followed by water (2 × 25 mL), dried over anhydrous Na_2_SO_4_, and concentrated. The residue was purified by column chromatography on silica gel, using light petroleum (distillation range 60–80 °C)–ethylacetate as eluent.

**Spectral data for selected compounds**

6-Butyl-1-methylpyrano[4,3-c]pyrazol-4(1*H*)-one (**3a**)

Light brown solid; mp 102–104 °C; ^1^H NMR (CDCl_3_, 400 MHz) δ 8.04 (s, 1H), 6.21 (s, 1H), 3.91 (s, 3H), 2.58 (t, *J* = 7.5 Hz, 2H), 1.73–1.68 (m, 2H), 1.44–1.39 (m, 2H), 0.95 (t, *J* = 7.5 Hz, 3H); IR cm^−1^ (KBr) 1730; *m/z* (CI Mass) 207 (M^+^+1, 100%); HPLC 98.5%, Inertsil ODS 3V (250 × 4.6) mm, mobile phase A: 0.01M KH_2_PO_4_ (pH 6.5), mobile phase B: CH_3_CN, gradient (*T*/% B): 0/30, 3/30, 14/80, 20/80, 21/30, 22/30, flow rate: 1.0 mL/min, UV 215 nm, retention time 8.3 min; Elemental Analysis found: C, 64.0; H, 6.80; N, 13.70 C_11_H_14_N_2_O_2_ Requires C, 64.06, H, 6.84, N, 13.58.

6-(2-Hydroxypropan-2-yl)-1-methylpyrano[4,3-c]pyrazol-4(1*H*)-one (**3e**)

Light brown solid; mp 140–142 °C; ^1^H NMR (CDCl_3_, 400 MHz) δ 8.09 (s, 1H), 6.67 (s, 1H), 3.95 (s, 3H), 1.59 (s, 6H); IR cm^−1^ (KBr) 3381, 1720; *m/z* (CI Mass) 209 (M+1, 100%); ^13^C (CDCl_3_, 50 MHz): δ 167.1, 157.9, 144.6, 138.1, 132.1, 105.7, 71.6, 36.1, 28.6 (2C); HPLC 98.3%, Inertsil ODS 3V (250 × 4.6) mm, mobile phase A: 0.01M KH_2_PO_4_, mobile phase B: CH_3_CN, gradient (*T*/% B): 0/20, 10/20, 15/70, 24/70, 26/20, 28/20, flow rate: 1.0 mL/min, UV 220 nm, retention time 8.1 min; Elemental Analysis found: C, 57.51; H, 5.69; N, 13.57 C_10_H_12_N_2_O_3_ Requires C, 57.68, H, 5.81, N, 13.45.

6-(3-Hydroxypropyl)-1-methylpyrano[4,3-c]pyrazol-4(1*H*)-one (**3f**)

Light brown solid; mp 124–126 °C; ^1^H NMR (CDCl_3_, 400 MHz) δ 8.05 (s, 1H), 6.28 (s, 1H), 3.92 (s, 3H), 3.73 (t, *J* = 6.2, 2H), 2.72–2.68 (m, 2H), 2.01–1.94 (m, 2H), 1.58 (bs, -OH); IR cm^−1^ (KBr) 3380, 1728; *m/z* (CI Mass) 209 (M+1, 100%); ^13^C (CDCl_3_, 50 MHz): δ 162.2, 158.1, 144.3, 137.2, 105.2, 91.2, 60.1, 38.7, 29.9, 29.5; HPLC 97.5%, Inertsil ODS 3V (250 × 4.6) mm, mobile phase A: 0.01 M KH_2_PO_4_, mobile phase B: CH_3_CN, gradient (*T*/% B): 0/15, 5/15, 15/70, 24/70, 26/15, 28/15, flow rate: 1.0 mL/min, UV 215 nm, retention time 10.4 min; Elemental Analysis found: C, 57.79; H, 5.80; N, 13.21 C_10_H_12_N_2_O_3_ Requires C, 57.68, H, 5.81, N, 13.45.

1-Methyl-6-phenylpyrano[4,3-c]pyrazol-4(1*H*)-one (**3g**)

White solid; mp 226–228 °C; ^1^H NMR (CDCl_3_, 400 MHz) δ 8.11 (s, 1H), 7.90–7.87 (m, 2H), 7.48–7.46 (m, 3H), 6.86 (s, 1H), 4.00 (s, 3H); IR cm^−1^ (KBr) 1725; *m/z* (CI Mass) 227 (M+1, 100%); ^13^C (CDCl_3_, 50 MHz): δ 157.9, 144.8, 138.2, 131.7, 130.9 (2C), 128.9 (2C), 125.7 (2C), 106.4, 89.4, 36.3; HPLC 98.1%, Inertsil ODS 3V (250 × 4.6) mm, mobile phase A: 0.01M KH_2_PO_4_ (pH: 6.5), mobile phase B: CH_3_CN, gradient (*T*/% B): 0/50, 5/50, 15/80, 24/80, 26/50, 28/50, flow rate: 1.0 mL/min, UV 210 nm, retention time 8.2 min; Elemental Analysis found: C, 68.81; H, 4.45; N, 12.51 C_13_H_10_N_2_O_2_ Requires C, 69.02, H, 4.46, N, 12.38.
